# Influencing factors and predictive model for left atrial appendage emptying velocity in nonvalvular AF patients

**DOI:** 10.3389/fcvm.2024.1468379

**Published:** 2024-09-19

**Authors:** Weibin He, Lei Yin, Qian Liu, Yan Zhang, Yanlei Zhao, Lianxia Wang, Ling You

**Affiliations:** Department of Cardiology, The Second Hospital of Hebei Medical University, Shijiazhuang, China

**Keywords:** atrial fibrillation, left atrial appendage emptying velocity, predictive model, echocardiography, retrospective study

## Abstract

**Background:**

Atrial fibrillation (AF) is the most common cardiac arrhythmia, significantly increasing the risk of death and stroke. The left atrial appendage (LAA) plays a crucial role in the development of AF. Reduced left atrial appendage emptying velocity (LAAEV) is an important indicator of nonvalvular AF, associated with thrombosis and recurrence after catheter ablation. This study aims to identify factors influencing LAAEV and construct a predictive model for LAAEV in nonvalvular AF patients.

**Methods:**

This retrospective cohort study included 1,048 nonvalvular AF patients hospitalized at the Second Hospital of Hebei Medical University from January 1, 2015, to December 31, 2021. Patients underwent transthoracic and transesophageal echocardiography and had complete laboratory data. Statistical analyses included binary logistic regression and multiple linear regression to identify independent predictors of reduced LAAEV and construct a predictive model.

**Results:**

Patients were divided into two groups: reduced LAAEV (<40 cm/s) and normal LAAEV (≥40 cm/s). The reduced LAAEV group included 457 patients (43.61%), with significant differences in age, gender, alcohol consumption, heart failure (HF), ischemic stroke, AF type, resting heart rate, CHA2DS2-VASc score, serum creatinine (SCR), serum uric acid (SUA), estimated glomerular filtration rate (eGFR), glycated hemoglobin (HbA1C), β2 macroglobulin (B2M), left atrial diameter (LAD), and left ventricular ejection fraction (LVEF) compared to the normal LAAEV group. Logistic regression analysis identified age (OR 0.974, 95% CI 0.951–0.997, *P* = 0.028), HF (OR 0.637, 95% CI 0.427–0.949, *P* = 0.027), AF type [Persistent AF vs. PAF (OR 0.063, 95% CI 0.041–0.095, *P* = 0) Long-standing Persistent AF vs. PAF (OR 0.077, 95% CI 0.043–0.139, *P* = 0)], LAD (OR 0.872, 95% CI 0.836–0.91, *P* < 0.001), and LVEF (OR 1.057, 95% CI 1.027–1.089, *P* = 0) as independent predictors of reduced LAAEV. Multiple linear regression analysis included age, AF type, LAD, and LVEF in the final predictive model, explaining 43.5% of the variance in LAAEV (adjusted R² = 0.435).

**Conclusion:**

Age, HF, type of AF, LAD, and LVEF are independent predictors of reduced LAAEV. The predictive model (LAAEV = 96.567–15.940 × AFtype–1.309 × LAD–0.18 × Age + 37.069 × LVEF) demonstrates good predictive value, aiding in the initial assessment and management of nonvalvular AF patients.

## Introduction

Atrial fibrillation (AF) is the most common cardiac arrhythmia (1), increases the risk of death by 1.5–1.9 times (2) and stroke risk by fivefold (3). The left atrial appendage (LAA) plays a crucial role in the development of AF. As a part of the left atrium (LA), the LAA has both contractile and endocrine functions, but it differs from the LA in developmental, structural, and physiological characteristics, contributing significantly to arrhythmogenesis and thrombosis (4). Approximately 90% of thrombi in nonvalvular AF patients originate from the LAA (5). Left atrial appendage emptying velocity (LAAEV) is an important indicator of nonvalvular AF, measured via transesophageal echocardiography, an invasive procedure (6). Decreased LAAEV is a known risk factor for LAA thrombosis in patients with nonvalvular AF (7). Catheter ablation is becoming the first-line treatment for nonvalvular AF (8). Decreased left atrial appendage emptying velocity is an independent predictor of recurrence after catheter ablation in patients with nonvalvular AF (9).

Several factors influence LAAEV. Previous studies have shown that LAAEV is related to the degree of LAA fibrosis (10), serum uric acid levels (11), nocturnal intermittent hypoxia (12), low voltage area in the anterior LA wall (13) and the morphology and opening area of the LAA (14). Retrospective studies identified non-paroxysmal AF, heart failure (HF), and age >65 years as predictors of LAAEV <20 cm/s and LAA thrombosis (15). Another retrospective study showed that LAAEV was not associated with LAA morphology and type of AF but with a higher CH2DS2 score and greater LAA volume (16). However, the sample size of studies on the factors influencing LAAEV is small and still controversial. Therefore, this study aims to investigate the potential factors influencing LAAEV and construct a predictive model for easier estimation of LAAEV, aiding in thromboprophylaxis and recurrence prevention post-catheter ablation in AF patients.

## Methods

This single-center retrospective cohort study collected laboratory tests and echocardiographic examinations of nonvalvular AF patients hospitalized at the Second Hospital of Hebei Medical University from January 1, 2015, to December 31, 2021, using the electronic medical record system.

Inclusion criteria for patients were as follows. (1) Age >18 years old. (2) Non-valvular AF type. (3) Underwent transthoracic and transesophageal echocardiography with complete laboratory data. The exclusion criteria were as follows. (1) Moderate or severe aortic or mitral stenosis. (2) History of surgery on the mitral or aortic valve. (3) Missing clinical data.

**Figure 1:**
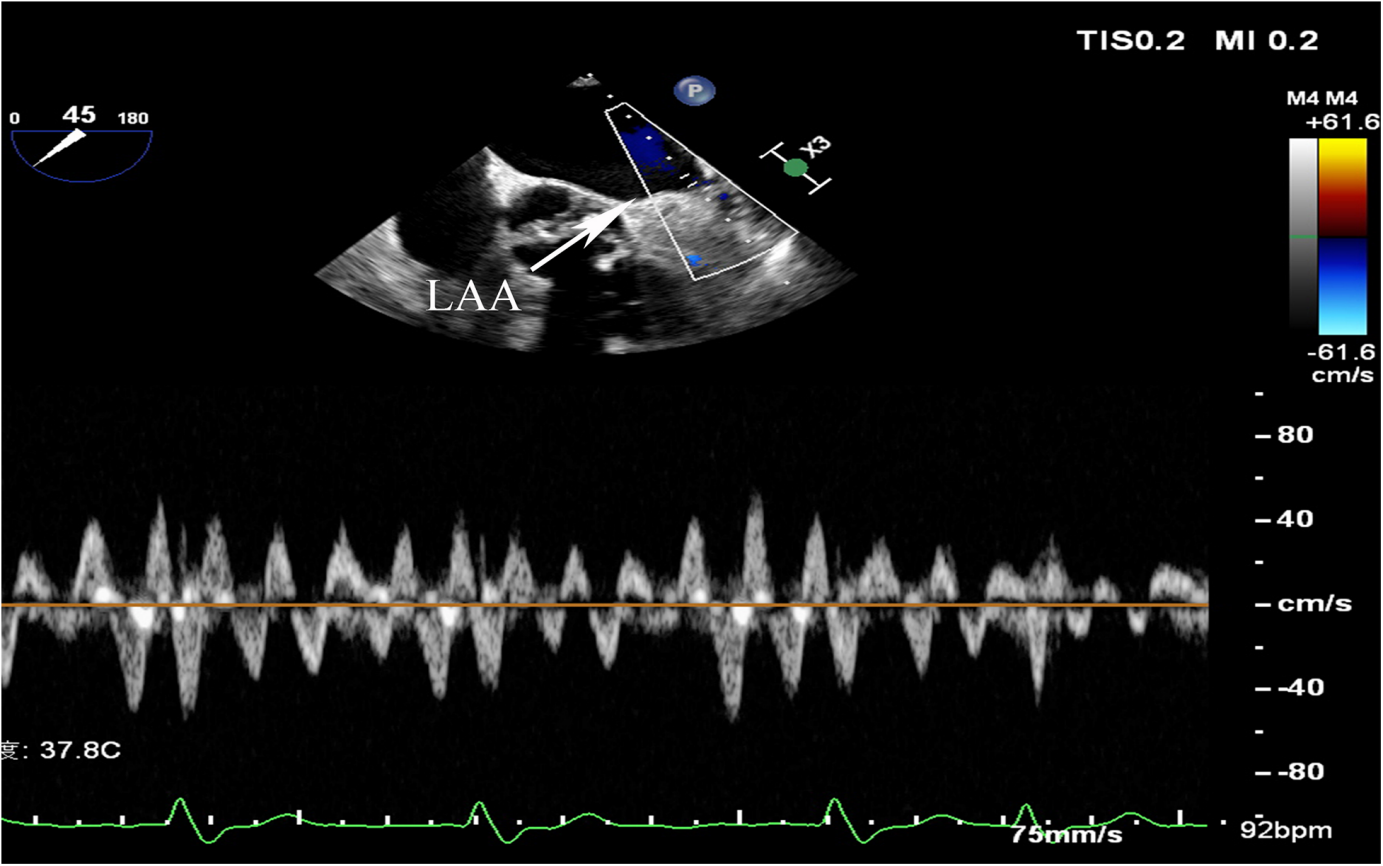
LAA, left atrial appendage.

AF was diagnosed based on electrocardiographic features of AF on a 12-lead electrocardiogram, a duration of AF >30 s on a 24-hour dynamic electrocardiogram, or a previous episode of AF. Paroxysmal AF(PAF) was defined as AF that terminated spontaneously or with intervention within seven days, persistent AF(PeAF) was defined as AF lasting more than seven days, and long-standing persistent was defined as continuous AF of >12 months’ duration (17). Diabetes mellitus (DM) was defined by random blood glucose ≥200 mg/dl, fasting blood glucose ≥126 mg/dl, or 2-hour blood glucose ≥200 mg/dl based on a 75 g oral glucose tolerance test, or the use of hypoglycemic medications or insulin. Hypertension was defined as systolic blood pressure ≥140 mmHg, or diastolic blood pressure ≥90 mmHg, or use of antihypertensive drugs. HF included past or current signs and symptoms of HF with both low (<40%) and preserved ejection fraction (≥40%) and other clinical evidence of cardiac dysfunction. Ischemic stroke was diagnosed based on CT or MRI evidence or a history of ischemic stroke. Peripheral arterial disease (PAD) was diagnosed via vascular Doppler ultrasound or past medical history. Coronary heart disease (CHD) was diagnosed according to relevant guidelines or history of CHD. Transthoracic echocardiograms and transesophageal echocardiograms were measured by an experienced sonographer and reviewed by an experienced senior sonographer. Left atrial diameters (LAD) were measured from the anteroposterior diameter of the LA, and left ventricular ejection fraction(LVEF) was measured using the modified Simpson method (18). LAAEV was obtained by transesophageal echocardiography and the pulsed doppler sample volume was typically placed 1–2 cm from the orifice within the chamber (6) (Figure 1). LAAEV of <40 cm/s were considered to be reduced (19, 20). LAAEV <20 cm/s were associated with embolic events (21). Therefore, a subgroup analysis was conducted. The estimated glomerular filtration rate (eGFR) was calculated by the CKD-EPI formula based on serum creatinine (SCr) (22). Patients’ CHA2DS2-VASc scores were calculated upon admission. The Clinical, imaging, and laboratory data were collected at the time of admission.

## Statistical analysis

Statistical analyses were performed using SPSS (version 26.0, SPSS Inc., Chicago, IL, USA). The Shapiro-Wilk test assessed whether continuous variables were normally distributed. Non-normally distributed continuous variables were expressed as medians and interquartile ranges, with the Mann-Whitney *U*-test used for sample comparisons. Categorical variables were presented as numbers and percentages, compared using chi-square tests. Binary logistic regression identified independent risk factors, with the odds ratio (OR) value and 95% confidence interval calculated. Multiple linear regression analysis, performed using stepwise regression with α_in_ ≤0.05 and α_out_ ≥0.1, constructed a predictive model. Pearson correlation analysis and Spearman correlation analysis in bivariate correlation analysis selected variables for the multiple linear regression model. Two-tailed *P*-values less than 0.05 were considered statistically significant.

## Result

Patients with non-valvular AF hospitalized at the Second Hospital of Hebei Medical University from January 1, 2015, to December 31, 2021, meeting the inclusion criteria, were included in this study. A total of 1,048 patients were analyzed. Patients were categorized into a reduced LAAEV group (LAAEV <40 cm/s) and a normal LAAEV group (LAAEV ≥40 cm/s). The reduced LAAEV group comprised 457 patients (43.61%), including 346 males (61.02%) and 221 females (38.98%). The normal LAAEV group consisted of 591 patients (56.39%), including 270 males (56.13%) and 211 females (43.87%) (Table 1).

**Table 1:** Baseline data.

Variable		LAAEV <40 cm/s (*n* = 457)	LAAEV ≥ 40 cm/s (*n* = 591)	*p*
Age, years old		64 (57–69)	62 (55–69)	0.008
Gender				0.038
	Male, *n* (%)	285 (62.36%)	331 (56.01%)	
	Female, *n* (%)	172 (37.64%)	260 (43.99%)	
Smoker, *n* (%)		101 (22.10%)	107 (18.10%)	0.108
Alcohol consumption, *n* (%)		90 (19.96%)	87 (14.72%)	0.033
Hypertension, *n* (%)		265 (57.99%)	335 (56.68%)	0.672
Diabetes, *n* (%)		87 (19.04%)	96 (16.24%)	0.237
HF, *n* (%)		279 (61.05%)	143 (24.20%)	0
PAD, *n* (%)		84 (18.38%)	107 (18.10%)	0.909
Ischemic stroke, *n* (%)		185 (40.48%)	156 (26.40%)	0
CHD, *n* (%)		155 (33.92%)	219 (37.06%)	0.293
AF type				0
	Paroxysmal AF, *n* (%)	85 (18.60%)	521 (88.2%)	
	Persistent AF, *n* (%)	257 (60.2%)	50 (8.5%)	
	Long-standing Persistent AF	97 (21.2%)	20 (3.4%)	
CHA2DS2-VASc score		3 (2–5)	3 (1–4)	0.001
Resting heart rate (bpm)		80 (70–94.5)	71 (61–82)	0
hs-CRP (mg/L)		1.5 (0.8–3.2)	1.4 (0.7–2.5)	0.054
SCr (μmmol/L)		73 (64–84)	69 (60–80.4)	0
eGFR,ml/min/1.73m2		88.01 (76.93–96.79)	92.45 (82.06–100.27)	0
HbA1C, %		5.9 (5.6–6.4)	5.8 (5.5–6.2)	0
B2M (mg/L)		1.9 (1.6–2.3)	1.8 (1.55–2.1)	0
SUA (μmmol/L)		335 (281–398.5)	304 (259–357)	0
TC (mmol/L)		3.95 (3.36–4.59)	4.01 (3.39–4.66)	0.428
TG (mmol/L)		1.22 (0.92–1.68)	1.26 (0.93–1.74)	0.488
LDL (mmol/L)		2.39 (1.92–3.02)	2.4 (1.89–3.07)	0.83
HDL (mmol/L)		1.1 (0.94–1.28)	1.12 (0.98–1.3)	0.111
APOA g/L		1.15 (0.97–1.31)	1.16 (1–1.34)	0.231
APOB g/L		0.82 (0.69–1.02)	0.81 (0.66–0.99)	0.23
LAD, mm		41 (38–44)	35 (33–38)	0
LVEF,%		60.7(55.55–63.08)	63.2(61.4–66.1)	0

HF, heart failure; PAD, peripheral vascular disease; CHD, coronary heart disease; AF, atrial fibrillation; SCr, serum creatinine; eGFR, estimated glomerular filtration rate; HbA1C, glycated hemoglobin; hs-CRP, high-sensitivity C-reactive protein; B2M, β2-microglobulin; SUA, serum uric acid; TC, total cholesterol; TG, triglyceride; LDL, low-density lipoprotein; HDL, high-density lipoprotein; APOA, apolipoprotein A; APOB, apolipoprotein B; LAD, left atrium diameter; LVEF, left ventricular ejection fraction; LAAEV; left atrial appendage emptying velocity.

### Baseline data

Patients with reduced LAAEV were older compared to those with normal LAAEV [64(57–69) vs. 62(55–69) *P* = 0.008] (unit, years old), had a higher percentage of males [285(62.36%) vs. 331 (56.01%) *P* = 0.038], had a faster resting heart rate [80(70–94.5) vs. 71(61–82) *P* = 0] (bpm) and had a higher rate of alcohol consumption [90(19.96%) vs. 87(14.72%) *P* = 0.033]. There was no statistically significant difference in the proportion of smokers between the two groups. In terms of underlying diseases, the reduced LAAEV group had a higher rate of HF [279(61.05%) vs. 143(24.20%) *P* = 0] and ischemic stroke [185(40.48%) vs. 156(26.40%) *P* = 0]. No statistically significant differences were observed in the prevalence of hypertension, DM, PAD, and CHD between the groups. In addition, the reduced LAAEV group also had a higher percentage of PeAF [257(60.2%) vs. 50(8.5%) *P* = 0] and long-standing persistent AF [97(21.2%) vs. 20(3.4%) *p* = 0], and a higher CHA2DS2-VASc score [3(2–5) vs. 3(1–4) *P* = 0.001].

In laboratory tests, transthoracic echocardiography and transesophageal echocardiography, compared to the normal LAAEV group, the reduced LAAEV group had higher levels of SCr [73(64–84) vs. 69(60–80.4) *P* = 0] (unit, mmol/L), glycated hemoglobin(HbA1C) [5.9%(5.6%−6.4%) vs. 5.8%(5.5%−6.2%) *P* = 0], β2 macroglobulin(B2M) [1.9(1.6–2.3) vs. 1.8(1.55–2.1) *P* = 0] (unit, mg/L), and serum uric acid(SUA) [335(281–398.5) vs. 304(259–357) *P* = 0] (unit, mmol/L). The reduced LAAEV group also had longer LAD [41 (38–44) vs. 35 (33–38), *P* = 0] (unit, mm), but lower eGFR [88.01 (76.93–96.79) vs. 92.45 (82.06–100.27), *P* = 0] (unit, ml/min/1.73m^2^) and LVEF [60.7% (55.55%−63.08%) vs. 63.2% (61.4%−66.1%), *P* = 0]. Differences in high-sensitivity C-reactive protein (hs-CRP), triglycerides, cholesterol (TC), low-density lipoprotein (LDL), high-density lipoprotein (HDL), apolipoprotein A (ApoA), and apolipoprotein B (ApoB) were not statistically significant between the two groups (Table 1).

### Logistic regression analysis

Based on the baseline data comparison, statistically significant differences were found in age, gender, alcohol consumption, HF, ischemic stroke, AF type, CHA2DS2-VASc score, resting heart rate, SCr, SUA, eGFR, HbA1C, B2M, LAD, and LVEF between the reduced and normal LAAEV groups. Ischemic stroke was excluded from the binary logistic regression analysis as it was not a factor in LAAEV. The CHA2DS2-VASc scores were also excluded because they include factors such as age, gender, HF, and ischemic stroke. eGFR was calculated from SCr values, so only eGFR was selected for inclusion in the binary logistic regression analysis. Since eGFR was calculated from SCr values, only eGFR was included in the binary logistic regression analysis. Thus, age, gender, alcohol consumption, HF, AF type, resting heart rate, eGFR, HbA1C, SUA, B2M, LAD, and LVEF were included in the binary logistic regression analysis.

Binary logistic regression results indicated that advanced age (OR 0.974, 95% CI 0.951–0.997, *P* = 0.028), suffering from HF (OR 0.637, 95% CI 0.427–0.949, *P* = 0.027), PeAF (PeAF vs. PAF (OR 0.063, 95% CI 0.041–0.095, *P* = 0), long-standing persistent AF (Long-standing Persistent AF vs. PAF (OR 0.077, 95% CI 0.043–0.139, *P* = 0), and lager LAD (OR 0.872, 95% CI 0.836–0.91, *P* = 0) were independent predictors of reduced LAAEV, while higher LVEF (OR 1.055, 95%CI 1.024–1.086 *P* = 0) were independent predictors of normal LAAEV. However, gender, alcohol consumption, eGFR, HbA1C, resting heart rate, SUA, and B2M were confounding factors (Table 2).

**Table 2:** Binary logistic regression results.

Variable		OR	95% CI	*P*
Age		0.974	0.951–0.997	0.028
Gender		0.882	0.538–1.257	0.336
Alcohol consumption		1.044	0.620–1.755	0.872
HF		0.637	0.427–0.949	0.027
AF Type				0
	Persistent AF vs. PAF	0.063	0.041–0.095	0
	Long-standing Persistent AF vs. PAF	0.077	0.043–0.139	0
Resting heart rate		0.994	0.986–1.003	0.216
eGFR		1.001	0.985–1.018	0.893
HBA1C		0.9	0.741–1.094	0.291
B2M		1.155	0.791–1.688	0.456
SUA		1	0.998–1.002	0.876
LAD		0.872	0.836–0.91	0
LVEF		1.055	1.024–1.086	0

HF, heart failure; AF, atrial fibrillation; eGFR, estimated glomerular filtration rate; B2M, β2-microglobulin; SUA, serum uric acid; LAD, left atrium diameter; LVEF, left ventricular ejection fraction; HbA1C, Glycated hemoglobin; PAF, paroxysmal AF.

### Multiple linear regression model of LAAEV

**Figure 2:**
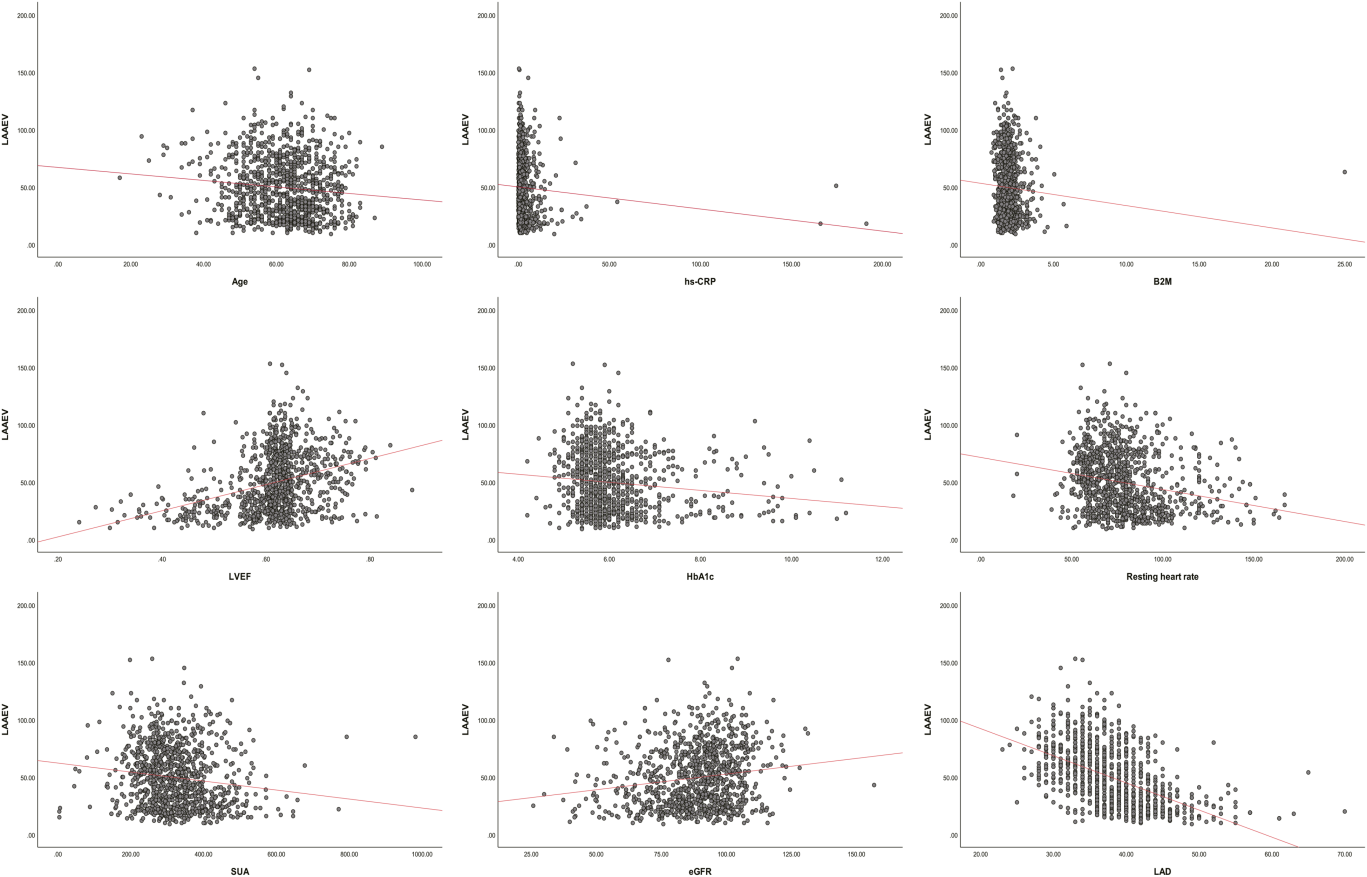
LAAEV, left atrial appendage emptying velocity; LVEF, left ventricular ejection fraction; SUA, serum uric acid; hs-CRP, high-sensitivity C-reactive protein; eGFR, estimated glomerular filtration rate; B2M, β2-microglobulin; LAD, left atrium diameter; HbA1C, glycated hemoglobin.

Correlation analysis indicated that age (r = −0.106, *P* = 0.001), alcohol consumption (r = −0.080, *P* = 0.01), AF Type (r = −0.661, *P* = 0), HF (r = −0.349, *P* = 0), resting heart rate (−0.220 *P* = 0), SUA (r = −0.14, *P* = 0), hs-CRP (r = −0.076, *P* = 0), HbA1C (r = −0.126, *P* = 0), B2M (r = −0.067 *P* = 0.029), and LAD (r = −0.513, *P* = 0) were negatively correlated with LAAEV, while eGFR (r = 0.162, *P* = 0) and LVEF (r = 0.333, *P* = 0) were positively correlated. Gender, Smoking, DM, hypertension, PAD, CAD, TC, TG, LDL, HDL, ApoA, and ApoB had no significant correlation with LAAEV (Supplementary Table 1). Scatter plots of age, hs-CRP, HbA1C, resting heart rate, SUA, B2M, LAD, eGFR, and LVEF vs. LAAEV demonstrated linear relationships (Figure 2).

Independent variables including age, alcohol consumption, AF type, HF, hs-CRP, HbA1C, resting heart rate, SUA, B2M, LAD, eGFR, and LVEF were selected for stepwise regression to construct a multiple linear regression model of LAAEV. After stepwise regression, age, type of AF, LAD, and LVEF were included in the regression model. The final constructed multiple linear regression model was statistically significant (F = 200.661 *P* = 0), and 43.5% of the variance in the dependent variable LAASE could be explained by changes in the type of AF, LAD, age, and LVEF (corrected R^2^ = 0.435). The regression coefficients (B) and 95% confidence intervals of the regression model are shown in Table 3. The regression equation is: LAAEV = 96.567–15.940 × AFtype–1.309 × LAD–0.18 × Age + 37.069 × LVEF, where PAF is coded as 0, PeAF as 1, Long-standing Persistent AF as 2, LAD is in millimeters, and age is in years.

**Table 3:** The regression coefficients (B) and 95% confidence intervals.

Variable	B	95.0% CI	*P*
AF Type	−15.940	−17.941–13.939	0
LAD	−1.309	−1.550–1.068	0
Age	−0.187	−0.310–0.063	0.003
LVEF	37.069	20.179–53.960	0
Constant	96.567	80.420–112.732	0

AF, atrial fibrillation; LAD, left atrium diameter; LVEF, left ventricular ejection fraction.

### Subgroup analysis

In the subgroup analysis, patients with LAAEV <20 cm/s were older [64(59–70) vs. 63(55–69) *P* = 0.031] (unit, years old), had a higher rate of HF [72(61.5%) vs. 350(37.6%) *P* = 0], a higher proportion of PeAF [80(68.4%) vs. 245(26.3%) *P* = 0] and long-standing persistent AF [24(20.5%) vs. 93(10%) *P* = 0], a higher CHA2DS2-VASc score [3(2–5) vs. 3(1–4) *P* = 0.001], and a faster resting heart rate [84(70–93.5) vs. 75(64–86) *P* = 0] (bpm). However, there were no statistically significant differences between the two groups in terms of gender, proportion of smokers, alcohol consumption, and the prevalence of hypertension, diabetes, PAD, ischemic stroke, and CHD. In terms of laboratory tests and ultrasound examinations, patients with LAAEV <20 cm/s had higher levels of hs-CRP [1.8(1–4.6) vs. 1.4(0.7–2.7) *P* = 0.001] (unit, mg/L), SCr [73(63.2–88) vs. 70(61–81) *P* = 0.032] (unit μmmol/L) and SUA [332(291.5–408) vs. 316(264–375) *P* = 0.008] (unit, μmmol/L) as well as larger LAD [42 (30–46) vs. 37 (34–40), *P* = 0] (unit,mm), while lower eGFR [86.72 (74.55–96.54) vs. 91.25 (81.09–99.14), *P* = 0.008] (unit, ml/min/1.73m^2^), TC levels [3.8(3.265–4.285) vs. 4(3.39–4.68) *P* = 0.034] (unit, mmol/L), and LVEF [58.33% (51%−62.885%) vs. 62.03% (60.23%−64.79%), *P* = 0] (Supplementary Table 2). Since the CHA2DS2-VASc score includes variables such as age and HF and eGFR is calculated from SCr, the CHA2DS2-VASc score and SCr were excluded from the binary logistics regression analysis. Ultimately, age, HF, AF type, resting heart rate, eGFR, SUA, TC, LAD and LVEF were included in the regression analysis. Binary logistic regression analysis showed that PeAF [PeAF vs. PAF (OR 0.119, 95% CI 0.061–0.229, *P* = 0], long-standing Persistent AF [Long-standing Persistent AF vs. PAF (OR 0.174, 95% CI 0.08–0.379, *P* = 0] and lager LAD(OR 0.886, 95% CI 0.851–0.923, *P* = 0) were independent predictors of LAAEV <20 cm/s, while higher LVEF(OR 1.029, 95% CI 1.002–1.085, *P* = 0.037) were independent predictors of LAAEV2 ≥20 cm/s (Supplementary Table 3).

## Discussion

This retrospective study analyzed non-valvular AF patients hospitalized at the Second Hospital of Hebei Medical University from January 1, 2015, to December 31, 2021, to identify factors influencing reduced LAAEV and construct a prediction model. The results indicated that age, HF, AF type, LAD, and LVEF are independent predictors of reduced LAAEV, and the constructed model has good predictive value.

Advanced age is a known independent predictor of cardiogenic stroke in patients with nonvalvular AF patients (23), and with a lower age threshold for stroke in Asian populations (24). In patients with PeAF, advanced age correlates with LAA thrombosis, which is less likely to resolve in older patients (25). Age remains an independent predictor of LAA thrombosis even after anticoagulation therapy (26). In healthy subjects, thrombus formation is detected in patients with lower LAAEV, and age is negatively correlated with LAAEV (27), corroborating our finding that higher age predicts lower LAAEV. In patients with nonvalvular AF, age-induced reduction in LAAEV appears to be an important reason why older patients are more susceptible to cardiac stroke. Aging is often accompanied by a decline in LA function (28), and increasing age is associated with a higher burden of LA fibrosis (29). In contrast, LA fibrosis strongly correlates with LAA fibrosis (30). This may be one of the reasons for the age-induced decrease in LAAEV, warranting further investigation.

HF is another independent predictor of cardiogenic stroke in patients with nonvalvular AF (23), and increases stroke risk even in sinus rhythm (31). In patients with nonvalvular AF with LAA thrombus, HF is not only an independent predictor of LAA thrombosis (32) but also an independent risk factor for the failure of the thrombus to resolve (33). Reduced LVEF also predicts LAA thrombosis in these patients (32). Our study confirmed HF and LVEF as independent predictors of reduced LAAEV, with LVEF negatively correlated with LAAEV. Patients with reduced LVEF or HF in nonvalvular AF are more likely to develop cardiac stroke or LAA thrombus, possibly related to the reduced LAAEV they cause. HF leads to decreased LA function, exacerbated in reduced ejection fraction HF (34). An animal experiment illustrated that HF can lead to remodeling and fibrosis of the LA (35). This explains, to some extent, the reduced LVEF in patients with HF and reduced LVEF, but further studies are needed.

PeAF is associated with more severe cardiac stroke (36). In addition, a real-world study showed that PeAF was independently associated with LAA thrombosis (37). PeAF patients have a higher stroke and systemic embolism risk than PAF patients, even with anticoagulation therapy (38). The greater susceptibility to LAA thrombus formation in patients with PeAF may be related to the reduced LAAEV. In this study, PeAF was an independent predictor of reduced LAAEV. A retrospective study showed that the extent of LA fibrosis assessed by gadolinium-enhanced MRI was greater in patients with PeAF than in PAF (39), which may contribute to their reduced LAAEV. The LAAEV serves as a predictive marker for the extent of LA fibrosis in patients with long-term persistent atrial fibrillation (40). A decreased LAAEV in these patients is potentially indicative of more advanced LA fibrosis. Still, the exact mechanism of reduced LAAEV in patients with Non-paroxysmal AF is unknown.

LA enlargement is more common in patients with AF, and AF is a cause of LA enlargement (41) and is also an independent predictor of stroke and systemic embolism in patients with nonvalvular AF (42). It is an independent predictor of recurrent cardiac stroke in the Chinese population (43). Retrospective studies had shown LA size as an independent predictor of reduced LAAEV (44)., with our study confirming this relationship. A study showed that LAD was independently associated with LA fibrosis in patients with nonvalvular AF (45). Patients with larger LAD have greater LA fibrosis, which may explain its association with reduced LAAEV.

LAAEV, measured via invasive transesophageal echocardiography, is crucial in nonvalvular AF (6). Our study provides a predictive model for LAAEV, with easily obtained variables like age, AF type, LAD, and LVEF, offering excellent predictive value for initial LAAEV assessment in nonvalvular AF patients, aiding in treatment evaluation.

This study demonstrated that age, AF type, HF, LAD, and LVEF independently predict reduced LAAEV, explaining the susceptibility to stroke in these patients. The predictive model has good value but requires further improvement. As a single-center retrospective study with patients from the Hebei region, findings may not generalize to other populations. Since this is a retrospective study, other indicators that may affect LAAEV, such as LA emptying fraction, LA volume, brain natriuretic peptide, brain natriuretic peptide and atrial fibrillation burden, were not available. So future research should include more variables and diverse populations to enhance the model’s predictive value.

## Conclusion

Age, heart failure, type of AF, LAD, and LVEF were identified as independent predictors of reduced LAAEV. The constructed model (LAAEV = 96.567–15.940 × AFtype–1.309 × LAD–0.18 × Age + 37.069 × LVEF) has demonstrated good predictive value, where PAF is coded as 0, PeAF as 1, Long-standing Persistent AF as 2, LAD is in millimeters, and age is in years.

## Data Availability

The raw data supporting the conclusions of this article will be made available by the authors, without undue reservation.
