# Supplementary material for: Influencing factors and predictive model for left atrial appendage emptying velocity in nonvalvular AF patients
Source: Front Cardiovasc Med. 2024 Sep 19;11:1468379. doi: 10.3389/fcvm.2024.1468379 (PMC11446783; doi:10.3389/fcvm.2024.1468379)
Supplement: Supplementary file 1 [file Table1.docx]

Table 1

| Variable | Correlation coefficient | P |
| --- | --- | --- |
| AF Type | -0.661 | 0 |
| Gender | -0.004 | 0.2 |
| Age | -0.106 | 0.001 |
| Smoking | -0.046 | 0.141 |
| Alcohol consumption | -0.080 | 0.010 |
| Diabetes | -0.046 | 0.134 |
| Hypertension | -0.020 | 0.507 |
| HF | -0.349 | 0 |
| PAD | -0.016 | 0.605 |
| CAD | 0.001 | 0.969 |
| Resting heart rate | -0.220 | 0 |
| hs-CRP | -0.076 | 0.014 |
| eGFR | 0.162 | 0 |
| HBA1C | -0.126 | 0 |
| B2M | -0.067 | 0.029 |
| SUA | -0.14 | 0 |
| TC | 0.039 | 0.204 |
| TG | 0.058 | 0.062 |
| LDL | 0.008 | 0.806 |
| HDL | 0.039 | 0.212 |
| APOA | 0.044 | 0.155 |
| APOB | -0.012 | 0.687 |
| LAD | -0.513 | 0 |
| LVEF | 0.333 | 0 |

HF: Heart failure PAD: Peripheral vascular disease CHD Coronary heart disease AF: Atrial fibrillation eGFR: Estimated glomerular filtration rate hs-CRP: High-sensitivity C-reactive protein B2M:β2-microglobulin SUA: Serum uric acid TC: total cholesterol TG: triglyceride LDL: Low-density lipoprotein HDL: High-density lipoprotein APOA: Apolipoprotein A APOB: Apolipoprotein B LAD: Left atrium diameter LVEF: Left ventricular ejection fraction LAAEV: Left atrial appendage emptying velocity. HbA1C: Glycated hemoglobin

| Variable |  | LAAEV＜20cm/s(n=117） | LAAEV≥20cm/s(n=931） | p |
| --- | --- | --- | --- | --- |
| Age,years old |  | 64(59-70) | 63(55-69) | 0.031 |
| Gender |  |  |  | 0.15 |
|  | male,n(%) | 76(65%) | 540(58%) |  |
|  | female,n(%) | 41(35%) | 391(42%) |  |
| Smoker,n(%) |  | 25(21.4%) | 183(19.7%) | 0.662 |
| Alcohol consumption,n(%) |  | 24(20.5%) | 153(16.4%) | 0.267 |
| Hypertension,n(%) |  | 66(56.4%) | 534(57.4%) | 0.845 |
| Diabetes,n(%) |  | 20(17.1%) | 163(17.5%) | 0.911 |
| HF,n(%) |  | 72(61.5%) | 350(37.6%) | 0 |
| PAD,n(%) |  | 24(20.5%) | 167(17.9%) | 0.496 |
| Ischemic stroke,n(%) |  | 45(38.5%) | 296(31.8%) | 0.147 |
| CHD,n(%) |  | 42(35.9%) | 332(35.7%) | 0.96 |
| AF type |  |  |  | 0 |
|  | Paroxysmal AF,n(%) | 13(11.1%) | 593(63.7%) |  |
|  | Persistent AF,n(%) | 80(68.4%) | 245(26.3%) |  |
|  | Long-standing Persistent AF | 24(20.5%) | 93(10%) |  |
| CHA2DS2-VASc score |  | 3(2-5) | 3(1-4) | 0.001 |
| Resting heart rate (bpm) |  | 84(70-93.5) | 75(64-86) | 0 |
| hs-CRP (mg/L) |  | 1.8(1-4.6) | 1.4(0.7-2.7) | 0.001 |
| SCR (μmmol/L) |  | 71(63.2-88) | 70(61-81) | 0.032 |
| eGFR,mL/min/1.73m2 |  | 86.72(74.55-96.54) | 91.25(81.09-99.14) | 0.008 |
| HbA1C,% |  | 5.9(5.6-6.45) | 5.8(5.5-6.3) | 0.07 |
| B2M (mg/L) |  | 1.9(1.6-2.3) | 1.81(1.6-2.16) | 0.54 |
| SUA (μmmol/L) |  | 332(291.5-408) | 316(264-375) | 0.008 |
| TC (mmol/L) |  | 3.8(3.265-4.285) | 4(3.39-4.68) | 0.034 |
| TG (mmol/L) |  | 1.17(0.89-1.64) | 1.25(0.93-1.74) | 0.242 |
| LDL (mmol/L) |  | 2.3(1.905-2.94) | 2.4(1.89-3.06) | 0.489 |
| HDL (mmol/L) |  | 1.06(0.905-1.25) | 1.12(0.97-1.29) | 0.067 |
| APOA g/L |  | 1.12(0.97-1.29) | 1.16(0.98-1.34) | 0.329 |
| APOB g/L |  | 0.8(0.68-1.01) | 0.82(0.67-1.01) | 0.885 |
| LAD,mm |  | 42(40-46) | 37(34-40) | 0 |
| LVEF,% |  | 58.33 (51-62.885) | 62.03(60.23-64.79) | 0 |

Table 2

Note: HF: Heart failure PAD: Peripheral vascular disease CHD Coronary heart disease AF: Atrial fibrillation SCr: Serum creatinine eGFR: Estimated glomerular filtration rate HbA1C: Glycated hemoglobin hs-CRP: High-sensitivity C-reactive protein B2M:β2-microglobulin SUA: Serum uric acid TC: total cholesterol TG: triglyceride LDL: Low-density lipoprotein HDL: High-density lipoprotein APOA: Apolipoprotein A APOB: Apolipoprotein B LAD: Left atrium diameter LVEF: Left ventricular ejection fraction LAAEV: Left atrial appendage emptying velocity.

Table 3

| Variable |  | OR | 95%CI | P |
| --- | --- | --- | --- | --- |
| Age |  | 0.977 | 0.95-1.005 | 0.113 |
| HF |  | 0.75 | 0.466-1.208 | 0.237 |
| AF Type |  |  |  | 0 |
|  | Persistent AF VS PAF | 0.119 | 0.061-0.229 | 0 |
|  | Long-standing Persistent AF VS PAF | 0.174 | 0.08-0.379 | 0 |
| Resting heart rate |  | 0.996 | 0.986-1.007 | 0.459 |
| eGFR |  | 1.002 | 0.986-1.019 | 0.796 |
| SUA |  | 1.001 | 0.998-1.003 | 0.668 |
| TC |  | 1.229 | 0.969-1.558 | 0.088 |
| LAD |  | 0.886 | 0.851-0.923 | 0 |
| LVEF |  | 1.029 | 1.002-1.085 | 0.037 |

Note: HF: Heart failure SUA: Serum uric acid TC: total cholesterol LAD: Left atrium diameter

LVEF: Left ventricular ejection fraction
